# A review of the amino acid metabolism in placental function response to fetal loss and low birth weight in pigs

**DOI:** 10.1186/s40104-022-00676-5

**Published:** 2022-03-02

**Authors:** Chengquan Tan, Zihao Huang, Wenyu Xiong, Hongxuan Ye, Jinping Deng, Yulong Yin

**Affiliations:** 1 Guangdong Provincial Key Laboratory of Animal Nutrition Control, National Engineering Research Center for Breeding Swine Industry, Institute of Subtropical Animal Nutrition and Feed, College of Animal Science, South China Agricultural University, Guangzhou, 510642 Guangdong China; 2 National Engineering Laboratory for Pollution Control and Waste Utilization in Livestock and Poultry Production, Institute of Subtropical Agriculture, Chinese Academy of Sciences, Changsha, 410125 Hunan China

**Keywords:** Amino acids, Birth weight, Fetal loss, Pigs, Placenta

## Abstract

The fertility of sows mainly depends on the embryo losses during gestation and the survival rate of the post-farrowing piglets. The selection of highly-prolific sows has been mainly focused on the selection of genotypes with high ovulatory quota. However, in the early- and post-implantation stages, the rate of embryo losses was increased with the increase of zygotes. Among the various factors, placental growth and development is the vital determinant for fetal survival, growth, and development. Despite the potential survival of fetuses with deficient placental development, their life-conditions and growth can be damaged by a process termed intrauterine growth retardation (IUGR). The newborn piglets affected by IUGR are prone to increased morbidity and mortality rates; meanwhile, the growth, health and welfare of the surviving piglets will remain hampered by these conditions, with a tendency to exacerbate with age. Functional amino acids such as glycine, proline, and arginine continue to increase with the development of placenta, which are not only essential to placental growth (including vascular growth) and development, but can also be used as substrates for the production of glutathione, polyamines and nitric oxide to benefit placental function in many ways. However, the exact regulation mechanism of these amino acids in placental function has not yet been clarified. In this review, we provide evidence from literature and our own work for the role and mechanism of dietary functional amino acids during pregnancy in regulating the placental functional response to fetal loss and birth weight of piglets. This review will provide novel insights into the response of nutritionally nonessential amino acids (glycine and proline) to placental development as well as feasible strategies to enhance the fertility of sows.

## Introduction

During the last two decades, the number of total born piglets per litter has been increased through genetic selection. Meanwhile, with the increase of litter size, the incidence of stillbirth increased and individual pig birth weight decreased, thus reducing the reproductive efficiency of sows [1, 2]. The largest contributor to a higher level of production is the number of live-born piglets of gilts or sows, which is directly affected by ovulation rate, fertilization rate, and conceptus survival to term. Sow is characterized with multi-ovulatory ovaries, ovulating approximately 20–30 follicles [3]. With a fertilization rate close to 90% [4], the number of early embryos can potentially reach 18–27. However, at farrowing, litter size is reduced to 9–13 piglets per sow due to the influence of many factors [5, 6]. One factor we must emphasize is the spontaneous conceptus loss. Two major waves of spontaneous embryonic or fetal loss have been documented in pigs: the first one occurs during the peri-attachment period (accounting for approximately 30% of total conceptus loss) and the second during mid-gestation (accounting for an additional 10–15% of conceptus loss) [7]. During the peri-attachment period (day 12–30 of pregnancy) for the attachment of conceptuses to the receptive endometrium [8, 9], variations in embryonic growth and elongation rates may alter the uterine environment, leading to lower survivability of less-developed conceptuses. During the mid-gestation period (day 50–70 of pregnancy), increased fetal growth may cause some conceptus attachment sites to exceed their uterine space, leading to conceptus arrest of adjacent littermates [8, 9].

Pigs exhibit the most severe (up to 25%) naturally occurring intrauterine growth retardation (IUGR) among livestock species [10, 11]. At present, IUGR piglets are culled on farms, and there is no nutritional support to increase their growth or survival during the suckling and postweaning periods. Moreover, large intra-litter variation in conceptus development is another major problem facing the global swine industry [12]. Birth weights of piglets can differ by up to three-fold in the same litter because the size, length and surface area of porcine placentae vary markedly among the conceptuses within the same uterus [13]. Heterogeneity in piglet weights among and within litters on the same farm increases the cost and challenge of managing a modern swine production system [14].

Studies over past few years have attempted to clearly delineate the causality related to spontaneous fetal loss and low weight of live-born piglets [4, 7, 15–17], which is attributed to uterine capacity [18], genetics [19], environment [20], immune mechanisms [21], and placental development [10, 22, 23]. However, the vital causes for porcine spontaneous conceptus loss and fetal growth retardation are still not well understood. The important contributors appear to be the placental development and function (deficits in vasculature) in early gestation and inadequate uterine capacity at all periods of gestation [10, 24, 25] rather than simply the number of ovulations or embryos [26].

## Early embryonic development and placentation in pigs

For the survival of conceptuses to term, we must coordinate the development of placentae and the specific adaptation of pregnancy to growth and development. For conceptus attachment and establishment or placental development, a series of events must proceed to ensure the uterine receptivity of the developing conceptuses, which include blastocyst hatching, hormone secretion, elongation into a filamentous conceptus and implantation and attachment [7, 15].

After fertilization, the zygote undergoes time-dependent mitotic divisions, resulting in embryos at different cleavage stages. A pig embryo enters the uterus between the four- to eight-cell stage and develops into a blastocyst shortly thereafter (around day 5). Between day 11–12 of pregnancy, the blastocyst secrets estrogen, changing the endometrial secretion of prostaglandin F2 alpha, allowing it to directly enter the uterine capillaries and release into the uterine lumen, eventually preventing the luteolysis of the corpus luteum and maintaining the progesterone secretion essential for a successful pregnancy [27, 28]. Between day 6 and 7 of pregnancy, the pig conceptus passes into the uterus as a spherical 100 mm blastocyst within a zona pellucida. At this stage, the development of pig embryos diverges dramatically from rodents or primates, and the presumptive placental membranes (trophectoderm and endoderm) elongate rapidly to a filamentous form by day 16 [15]. Placentation in the pig relies on conceptus elongation to increase the available surface area for gas and nutrient exchange [15].

Implantation is the process for the attachment of the blastocyst to the uterus for the juxtaposition of embryonic and maternal circulation, leading to the establishment of a functional placenta and successful pregnancy, with the initial attachment process starting around day 12 during gestation [29]. Peri-implantation conceptus elongation and implantation in pigs is modulated by the secretion of the endometrium in response to factors from the ovary (progesterone), the conceptus (estrogen, interleukin-1beta, interferons delta and gamma, and transforming growth factor beta) and endometrium (transforming growth factor beta and fibroblast growth factor 7) [15]. These complex events are orchestrated through, among others, four important cell signaling pathways for endocrine, paracrine, autocrine, and juxtracrine communication between conceptus and uterus, and deficiencies of these events are reflected by a high rate of conceptus mortality during the peri-implantation period of pregnancy [30]. Particularly, further production of oestrogen by the blastocyst within day 15–30 of pregnancy stimulates the secretion from the uterine epithelium and glands [27], leading to the production of specific proteins which are thought to be involved in the growth of the conceptus and the subsequent immunological readjustment necessary for successful implantation [31]. Collectively, communication and crosstalk between the peri-attachment conceptus and the endometrium are important for survival and placentation.

Between day 15 and 20 of pregnancy, trophoblastic and uterine membranes come close together, enabling the interdigitating microvilli to develop between the apical domes of the uterine epithelium and the trophoblast to cover the entire placenta except for the openings of uterine glands, leading to the transition from primarily histotrophic to hemotrophic nutrition [32]. Along the uterine gland openings, simple structures called areola [33] begin to form at day 25–30 of pregnancy while dome-shaped structures over the openings of uterine glands and the numbers are maximized by day 70 of gestation [34].

Placentae develop earlier than fetuses in gilts. Between day 20 and 60 of pregnancy, the porcine placenta begins to grow rapidly, with a significant increase in weight, size and surface area, followed by near-maximum development and vascularization at day 70 of pregnancy [7, 35]. During the development of placenta, the demand for amino acids increases gradually, and the placental total mass of amino acids increases linearly as gestation progresses [10, 36]. Glycine, proline, arginine, and alanine, rather than other amino acids, contribute to the increase of total amino acids with the progression of pregnancy, especially glycine and proline [10, 36]. The concentrations of proline in porcine placenta increase between day 20 and 40 of pregnancy, remain high until day 60 of gestation, and decline thereafter, which is consistent with the rapid growth of placenta in the first trimester of pregnancy [37]. These results suggest that the maturation and development of placenta may require more such amino acids to continue the synthesis of connective tissues and collagen, as well as support cell steady proliferation and angiogenesis. Moreover, in contrast to the placenta whose weight tends to stabilize, the growth rate of fetuses is very high after day 60 of gestation [38], indicating that the increased placental weight might promote fetal growth. Placental vascularization and angiogenesis are necessary for the marked increase of the utero-placental blood flow during this period to supply nutrients from mother to fetus for optimal growth [22, 39].

## Morphology and structure of the porcine placenta

**Fig. 1:**
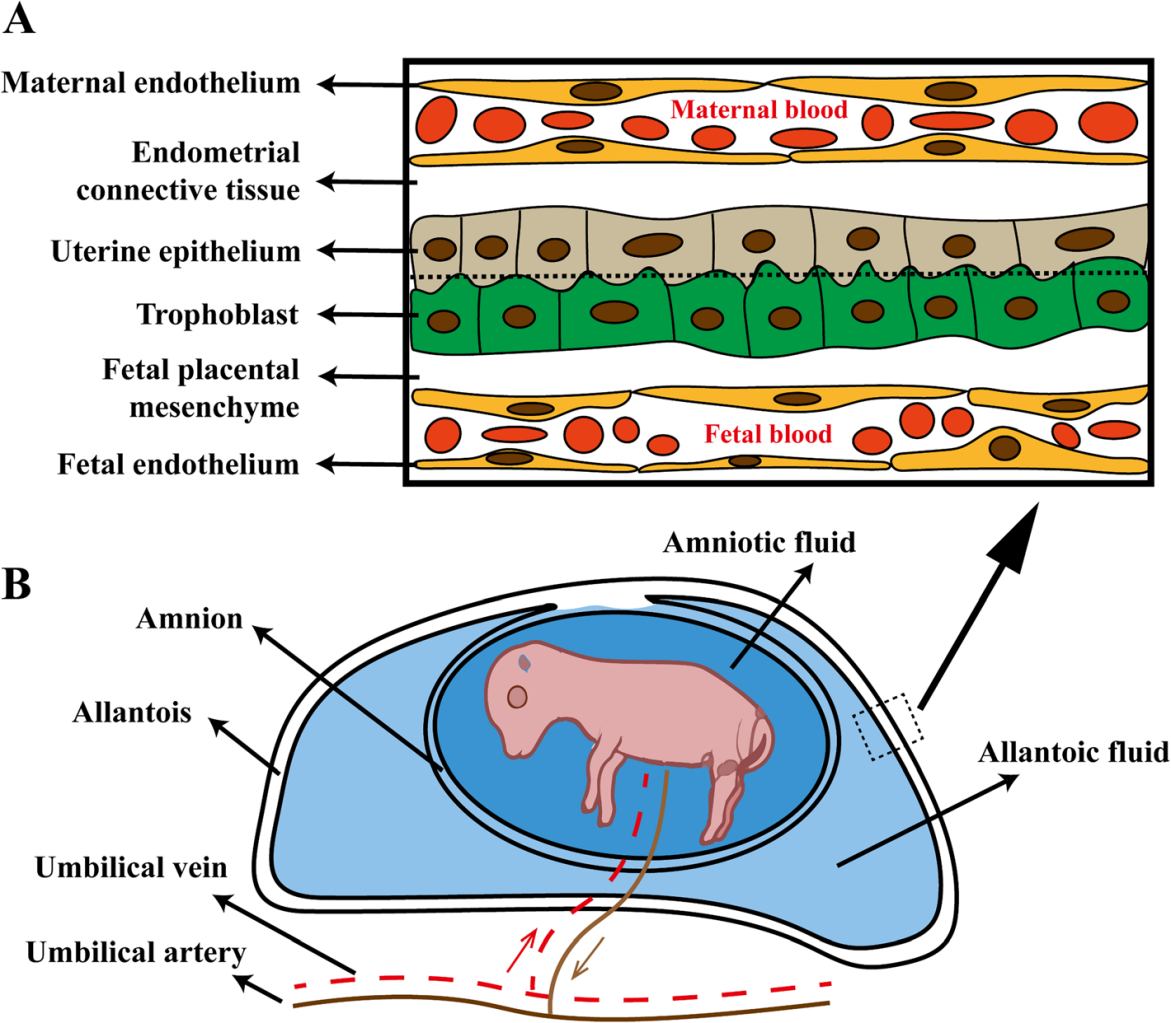
Porcine placental barrier and conceptus. Representations of the placental barrier in swine (A) and conceptus within the uterine horn (B). Note that the allantois also supports the vascular elements of the epitheliochorial placenta of the pig. The amnion is filled with amniotic fluid that facilitates the embryo/fetus to allow it to develop symmetrically and not adhere to other tissues

The swine placenta is a typical diffuse epitheliochorial organ without invasion [40], i.e., with neither invasion of fetal tissue into the maternal endometrium nor the occurrence of endometrial decidualization. Almost the entire surface of the allantochorion (embryonic membrane consisting of a fused allantois and chorion) is involved in the formation of placenta. Porcine fetuses have individual fetal membranes, and on day 39–55 of gestation, large placental zones of the individual conceptuses are terminated by the two extremities of the fetal sacs that include para-placental and ischemic zones (necrotic tips) [41]. Maternal and fetal microvilli appose and interdigitate, with a clear distinction between maternal and fetal tissues (semi-placenta). Meanwhile, maternal and fetal blood is separated by six tissue layers (Fig. 1) to form a firm barrier, which can even prevent maternal antibodies from passing through fetuses during gestation [42]. Since no invasion occurs, much of the placenta/embryonic development depends on the uterine milk or embryotroph (areolae). Interestingly, although the ungulate placenta is epitheliochorial, the placental barrier in certain regions is thinner than that found in carnivores with the endotheliachorial placenta (in this type of placenta, the endometrial epithelium under the placenta does not survive implantation, and fetal chorionic epithelial cells come in contact with maternal endothelial cells) [43]. Moreover, although epitheliochorial placentae are multilayered, the distance between fetal and maternal blood is minimized by the indentation of maternal capillaries into the uterine epithelium and fetal capillaries into the trophoblast [31, 33]. Another important advantage of epitheliochorial placentation is the detachment of the placenta at parturition with minimal damage to the uterus, which may accelerate the recovery of postpartum receptivity of the uterus as seen in pigs and horses [33].

## Role of amino acids in regulating placental function in sows

Among the nutrients required by pregnant sows, amino acids play the most important role in placental growth due to their involvement in the synthesis of sufficient proteins to support the proliferation of trophectoderm and endothelial cells [10]. Amino acids are not only the building blocks of proteins in cells, but also precursors for synthesis of nitrogenous substances (e.g., nitric oxide (NO), polyamines, and glutathione) essential for placental growth and angiogenesis, conceptus development or scavenging free radicals to prevent placental oxidative damage [10, 44, 45]. As mentioned earlier, the placental demand of amino acids varies markedly with the extension of gestation, so the supply of amino acids to the placenta must be controlled to ensure an adequate supply of each amino acid. Among all the amino acids measured in the placenta, glycine is the highest in content, followed by glutamate, proline, and arginine on day 60 of gestation [10]. Alanine, aspartate, asparagine, glutamate, and glutamine are synthesized from branched-chain amino acids and glucose in the porcine placenta [46]. As little is known about the de novo synthesis of glycine and proline in the placenta currently, this review mainly focuses on the roles of glycine, proline, and arginine in the regulation of placental function.

### Glycine

Glycine, the most abundant amino acid in oviductal, uterine and follicular fluids, plays a vital role during oocyte maturation, fertilization, and early embryonic development in pigs [47]. The catabolism of glycine provides a methyl group to folic acid for DNA synthesis and methylation of homocysteine [48]. In this pathway, the glycine cleavage system is catalyzed by a complex of four proteins, and among them, T-protein requires tetrahydrofolate as a cofactor and induces the formation of ammonia and 5,10-methylene-tetrahydrofolate [49]. In sows, glycine is the most abundant amino acid in the oviduct and uterine fluids during diestrus [50] and in the allantoic fluid on day 30 of gestation [51]. Therefore, the metabolic provision of glycine to uterus should be adequate for an optimal folate response. Indeed, dietary supplementation of folic acid + glycine is shown to optimize embryo development in multiparous Yorkshire-Landrace sows [52]. Furthermore, glycine supplementation rather than urea or alanine supplementation is found to play an important role in maternal S-amino acid metabolism in pregnancy and fetal cardiovascular development [53]. Compelling evidence shows that glycine could rectify vascular dysfunction induced by dietary amino acid imbalance during pregnancy [54].

Oxidative stress can impair the female reproductive physiology by altering the affectivity of antioxidant defense system in humans and animals. There is growing evidence that pregnant sows suffer from elevated levels of oxidative stress in late pregnancy and lactation [55–57], due to generation of reactive oxygen species that exceeds the capacity of antioxidant system [58]. Elevated levels of oxidative stress during gestation can impair placental function, including the angiogenesis [23, 59] and reproductive performance of sows [23, 60]. Previous study has shown that glycine is the precursor for the synthesis of glutathione, the most-abundant low-molecular-weight antioxidant in animal tissues, including placenta [61].

Available evidence shows that the amount of glycine synthesized in vivo is insufficient to meet the metabolic demands (i.e., the synthesis of proteins, glutathione, and heme) in humans, pigs, rodents, and other mammals, thus unable to support their maximal growth [62, 63]. It is worth noting that fetuses and neonates cannot synthesize enough glycine to meet their optimal requirements [64, 65], and chronic insufficiency of glycine may result in suboptimal growth, impaired immune responses, and other adverse effects on their health and nutrient metabolism [66, 67]. A study on the supplementation of 800 mg/kg N-carbamylglycinate (a derivative of glycine to improve glycine concentrations via the hydrolysis of an amide bond) in the diet of late-gestation sows showed that the nutritional levels of glycine and proline in sows were improved, and the litter size and litter weight of live-born piglets were increased [68]. All in all, the multiple beneficial effects of glycine, coupled with its insufficient de novo synthesis, support the notion that, as it cannot be adequately absorbed from the feed, glycine is both a conditionally essential and a functional amino acid for pregnant sows that need sufficient nutrients to support fetal growth [22, 61, 64, 69, 70]. Appropriate supplementation of glycine or its derivatives to pregnant sows may bring greater benefits to actual production, but this information is still limited and needs to be further enriched.

### Proline

**Fig. 2:**
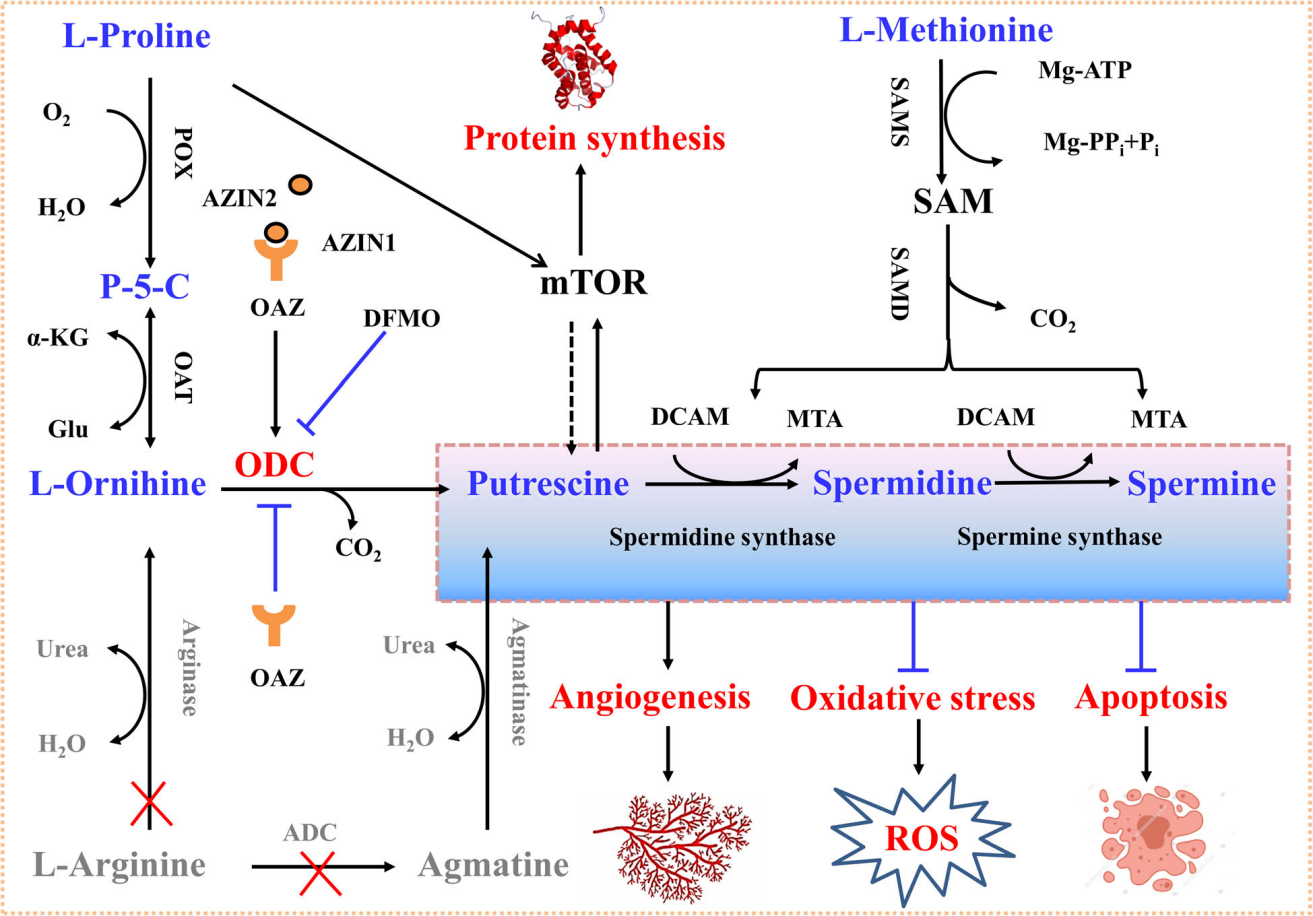
Synthesis of polyamines from proline and their function in the porcine placenta. Unlike most other tissues, which convert arginine to polyamines via arginase and ornithine decarboxylase, the porcine placenta lacks arginase activity, so it cannot synthesize ornithine from arginine. Degradation of ODC is regulated via OAZ, which binds to ODC, while AZIN, a protein with a similar structure to ODC but with no activity, can bind to OAZ with a higher affinity, thus preventing ODC degradation. Note that there are three isoforms of OAZ (OAZ1, OAZ2 and OAZ3) and two isoforms of AZIN (AZIN1 and AZIN2). DFMO is a catalytic, irreversible inhibitor of ODC. For porcine placentae, polyamines mainly have roles in angiogenesis, oxidative stress, protein synthesis, and apoptosis. Solid black arrows indicate direct activation, and dashed black arrows indicate indirect activation. ADC, arginine decarboxylase; α-KG, α-ketoglutarate; AZIN, antizyme inhibitor; DCAM, decarboxylated S-adenosylmethionine; DFMO, difluoromethylornithine; MTA, methylthioadenosine; mTOR, mechanistic target of rapamycin; OAT, ornithine aminotransferase; OAZ, antizyme; ODC, ornithine decarboxylase; P-5-C,△^1^-L-Pyrroline-5-Carboxylate; POX, proline oxidase; PP_i_, inorganic pyrophosphate; ROS, reactive oxygen species; SAM, S-adenosylmethionine; SAMD, S-adenosylmethionine decarboxylase; SAMS, S-adenosylmethionine synthase

Proline serves not only as the building block of proteins, but also as a nitrogenous substrate for endogenous synthesis of arginine, glutamate, and polyamines in mammals [37]. Especially, polyamines (e.g., putrescine, spermidine, and spermine) are key regulators of DNA and protein synthesis, cell proliferation, and differentiation in both the small intestine and placenta of mammals, including pigs [10, 63]. For porcine placentae, we focus on the function of polyamines in angiogenesis, oxidative stress, protein synthesis, and apoptosis (Fig. 2). Angiogenesis is the physiological process of generating new blood vessels from existing blood vessels in placenta and is the sum of important cellular events including migration, proliferation and growth of endothelial cells [71]. As polyamines can stimulate tissue growth, including placentae, an irreversible inhibitor of ornithine decarboxylase (alpha-difluoromethylornithine, DFMO) was developed to reduce polyamine synthesis at the cellular level [72]. As mentioned above, increased metabolic burdens on sows during late gestation and lactation tend to cause elevated systemic oxidative stress during these important periods [56, 60, 73]. Polyamines are potent antioxidants with cell-protective effects, because they can control the free radicals generated during cellular metabolisms, such as preventing structural and functional damage to DNA by blocking free radicals from binding [44]. Likewise, polyamines can inhibit lipid peroxidation in cell membranes to protect the functionality and structure of lipids in the cell membrane [74]. However, the roles of polyamines in scavenging free radicals remain unclear.

Previous study has indicated that supplementation with putrescine promotes the proliferation of porcine trophectoderm cells by increasing protein synthesis via activation of the mechanistic target of the rapamycin (mTOR) signaling pathway [75]. However, few studies have focused on the effect of putrescine on the reproductive performance of gilts or sows. We suggest that dietary supplementation with or intravenous administration of putrescine could be a potential novel and effective strategy to improve the survival and growth of embryos/fetuses in pigs. Additionally, in porcine parthenote embryos, the combined addition of all the three polyamines (putrescine, spermidine, and spermine) could increase the total cell number and blastocyst formation percentage as well as inhibit apoptosis by partially decreasing the expression of apoptotic genes [69, 76]. These effects of putrescine seem to be transduced through mTOR signaling because inhibiting mTOR with rapamycin prevents the above effects in the pig (Fig. 2) and reduces the level of interferon-τ induced by putrescine in the sheep conceptus [75, 77]. The above-mentioned facts indicate that polyamines have many important functions. One point we must emphasize is that the porcine placenta lacks arginase and arginine decarboxylase, thus unable to synthesize ornithine (the precursor for the synthesis of polyamines) from arginine [78]. Previous study has shown that the substrates for the synthesis of polyamines in the porcine placenta, and novel findings provide that proline oxidase is a rate-controlling enzyme in placental synthesis of polyamines, which converts proline to pyrroline-f-carboxylate, a precursor of both ornithine and polyamines [78]. Indeed, proline provides the bulk of the carbon skeleton and nitrogen in putrescine, spermidine, and spermine [10].

Moreover, compelling evidence from animal studies shows that reduced placental and fetal growth is associated with a reduction in placental proline transport in gestating dams with either naturally occurring or malnutrition-induced growth retardation [37]. During the late gestation period, reduced protein levels in diet may result in insufficient maternal dietary intake of protein and a decrease in the amino acid availability to the fetus, thereby retarding fetal growth [79, 80]. The amino acid and endogenous protein losses are mainly caused by high losses of glycine, proline, and serine [81]. In the amino acid composition of fetal pigs during the gestation period, glycine and hydroxyproline increased markedly while other amino acids decreased from day 60 to 114 of gestation [82]. However, the activities of proline oxidase, ornithine aminotransferase, and ornithine decarboxylase, as well as proline transport, polyamine synthesis from proline, and polyamine concentrations in placentae, increase markedly between day 20 and 40 of gestation, decline between day 40 and 90 of gestation, and remain at the reduced level from day 110 of gestation [78]. These results indicated that proline ingestion is insufficient during early gestation, which can be well supported by the study of Gonzalez-Anover et al., who demonstrated that the effects of the L-proline supplementation of 14 g per sow per day in early pregnancy on litter size and birth weight are strongly modulated by the maternal characteristics, such as parity and prolificacy, and that such supplementation can be cost-efficient for the management of females with compromised energy balance, such as sows at second farrowing and highly-prolific primiparous gilts [83]. Recent studies have indicated that maternal L-proline supplementation can improve placental development and fetal survival by enhancing placental nutrient transport, angiogenesis, and protein synthesis in F0 females, and increase the concentrations of polyamines in placental tissues in their F1 females of mice [84, 85].

### Arginine

Arginine serves as the physiological precursor for synthesis of protein and other biological molecules, including ornithine, polyamines, proline, glutamine, creatine, and NO [70]. Arginine and its metabolites have versatile functions in cardiovascular, neurological immunological and endocrine systems [86]. Here, we mainly focus on the effect of arginine nutrition on porcine embryonic, placental, and fetal development. In the past three decades, accumulated evidence has indicated that supplementation of arginine improves the reproductive performance in sows (Table 1). Arginine was remodeled as a nutritionally essential amino acid for gestating pigs in the revised version of National Research Council (NRC, 2012). However, previous studies have also reported that dietary arginine showed no or little effect on litter size and could even impair the reproductive performance of gilts and sows [87–89], suggesting that the dose and duration of supplementation should be considered in dietary formulations. Moreover, these results underscore the importance of understanding the basic knowledge of reproductive biology, arginine biochemistry and nutrition to improve the reproductive performance of sows.

**Table 1:** Summary of relevant reports on the effects of maternal dietary arginine supplementation on the reproductive performance in swine in the last decade

Supple-mentation period	Arginine content in basal diet, %	Supple-mental arginine, %	Litter size of live-born piglets	Litter weight of live-born piglets	Placental weight	References
d 14–25	0.70%	0.4%	↑by 2.2 per litter	–	↑by 34%	[110]
d 14–25	0.70%	0.8%	↑by 1.7 per litter	–	↑by 21%	[110]
d 14–28	1.07%	0.87%	↑by 3.7 per litter	↑by 32%	–	[111]
d 1–114	0.72%	0.25%	↑by 0.8 per litter	–	–	[112]
d 22–114	0.88%	0.83%	↑by 1.1 per litter	↑by 11%	↑by 16%	[113]
d 30–90 or d 30–114	0.73%	1%	↑by 1.6 per litter	↑	Un	[114]
d 30–110	0.61%	0.1%	↑by 1.1 per litter	–	–	[109]
d 70–110	0.72%	0.28%	↑by 1.2 per litter	–	–	[115]
d 70–110	0.72%	0.78%	↑by 1.1 per litter	↑by 12%	–	[115]
d 85–114	Un	1%	↑by 1.4 per litter	–	Un	[116]
d 90–114	Un	1%	–	↑by 16%	Un	[106]

Un, undetermined; ↑, increase; —, no effect

Generally, pregnant pigs are fed a restricted diet during gestation to avoid excess weight gains and associated farrowing and locomotion issue, and reduce lactational feed intake [90–93]. Thus, dietary provision of arginine for gestating sows is insufficient to meet arginine requirements. In gilts or sows, arginine in circulating blood is derived from diets and endogenous sources, including both de novo synthesis and protein degradation [70]. It is worth noting that only 60% of dietary arginine enters the portal circulation of pregnant gilts due to the extensive catabolism of arginine by arginase in the small intestine [70]. Additionally, approximately 8% of arginine in the portal vein is extracted by the liver for protein and urea synthesis [94]. Based on the actual ileal digestibility (85%) of arginine in a corn-soybean meal-based diet, only 46.92% of protein-bound arginine in the diet can be used by extra-intestinal and extra-hepatic tissues in gestating gilts [70]. The above-mentioned facts indicate the necessity for the endogenous synthesis of arginine to support embryonic, placental and fetal growth.

**Fig. 3:**
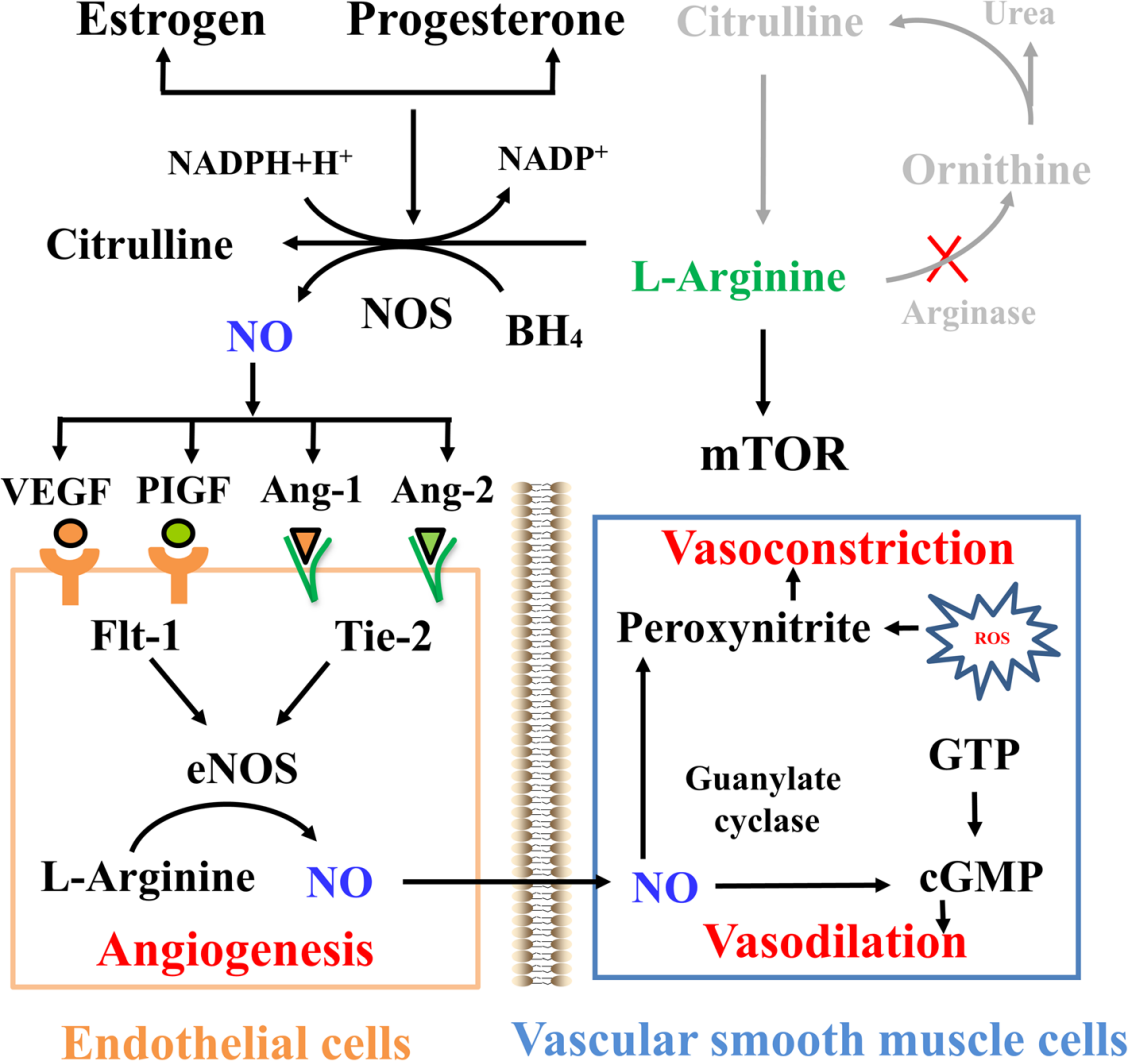
The L-arginine-NO biosynthetic pathway regulates key vasculogenic and angiogenic factors in endothelial cells and vascular smooth muscle cells in the placenta. Arginine stimulates placental NO synthesis mainly by enhancing the generation of BH_4_, an essential cofactor for NOSs. Estrogen and progesterone augmented the constitutive and inducible NOS activities and the BH_4_ concentration in the porcine placenta, thus playing synergetic roles in promoting the placental NO synthesis. The porcine placenta lacks arginase and arginine decarboxylase and thus cannot synthesize ornithine from arginine. NO interacts with critical mediators of angiogenesis (VEGF, PIGF, and the angiopoietins). The induction of angiogenesis by angiopoietin and VEGF proteins is dependent on the amount of VEGF and Ang-1/Ang-2 in endothelial cells. Besides, NO diffuses to the underlying VSMCs where they act on GTP to produce cGMP via guanylate cyclase, which is capable of dilating blood vessels. Ang-1/Ang-2, angiopoietin-1 and angiopoietin-2; BH_4_, tetrahydrobiopterin; cGMP, cyclic guanosine monophosphate; eNOS, endothelia nitric oxide synthase; Flt-1, fms-like tyrosine kinase-1; GTP, guanosine; mTOR, mechanistic target of rapamycin; NADP^+^, nicotinamide adenine dinucleotide phosphate; NADPH, nicotinamide adenine dinucleotide phosphate hydrogen; NO, nitric oxide; NOS, nitric oxide synthase; PIGF, placental growth factor; ROS, reactive oxygen species; Tie-2, tunica interna endothelia cell kinase 2; VEGF, vascular endothelial growth factor; VSMCs, vascular smooth muscle cells

Arginine is a common substrate for the synthesis of NO and polyamines, both of which affect angiogenesis and thus fetal growth. However, the porcine placenta lacks arginase and arginine decarboxylase and therefore cannot synthesize ornithine from arginine [78]. Arginine stimulates placental NO synthesis mainly by enhancing the generation of tetrahydrobiopterin (BH_4_) as an essential cofactor for all NO synthetases (NOSs) [22]. NO synthesis and arginine transport in cultured placentae showed a 6.3- and 6.7-fold increase between day 20 and 40 of gestation, respectively, followed by a decrease, suggesting the rapid decrease of arginine and NO to support angiogenesis in gestating swine [95]. Of particular note estrogen and progesterone enhanced the constitutive and inducible NOS activities as well as the BH_4_ concentration in the porcine placenta, thus playing a synergistic role in promoting placental NO synthesis [10]. The arginine-NO pathway is an important regulator of vascular development. At the endothelial interface, NO regulates critical mediators of angiogenesis, including the vascular endothelial growth factor (VEGF) (e.g., placental growth factor, its receptor fms-like tyrosine sinase-1) and angiopoietin-tunica interna endothelial cell kinase 2 (Tie-2) protein families (e.g., angiopoietin-1, angiopoietin-2 and their receptor). The induction of angiogenesis by angiopoietin and VEGF signaling is dependent on the protein-kinase-mediated downstream activation of endothelial NOS in the endothelium [96], and the consequent production of NO from arginine [97, 98]. Additionally, NO diffuses to the underlying vascular smooth muscle cells to act on guanosine and produce cyclic guanosine monophosphate via guanylate cyclase, which is capable of dilating blood vessels (Fig. 3) [99]. Additionally, arginine activates the mTOR cell signaling pathway to stimulate protein synthesis in the placenta, uterus, and fetus [100]. Collectively, the available evidence implicates the dysregulation of arginine-NO biogenesis in a range of vascular pathologies in humans and pigs [70, 101].

It has been widely accepted that arginine promotes the growth of skeletal muscle and reduces the accretion of white adipose tissue [102]. Apart from arginine play a role in angiogenesis of placental development, recent years have witnessed increasing interest in its effect on fetal myofiber formation during gestation as evidenced by a study reporting that arginine supplementation to sows during early gestation obviously enhanced not only prenatal myofiber hyperplasia but also hypertrophy [103]. In contrast, decreased availability of both arginine and NO increases the proliferation of preadipocytes and adipocytes in IUGR fetuses. Collectively, arginine regulates nutrient partitioning to promote skeletal muscle growth over white-fat accretion during gestation [22].

As an endogenous activator of arginine, N-carbamylglutamate (NCG) also has a similar effect on fetal growth and placental development. NCG, a safe and metabolically stable functional analog of N-acetylglutamate, can activate the key enzyme (carbamylphosphate synthetase-1) of the arginine-synthetic pathway and increase the endogenous synthesis of arginine [104, 105]. Studies have shown that both arginine and NCG can increase litter size and fetal birth weight of sows, which may be related to the up-regulation of placental angiogenesis [106], and similar results have been reported in pregnant ewes [107]. The results in pregnant rats confirm that NCG can effectively increase the concentration of arginine family amino acids in maternal circulation and uterine fluid, thus improving the pregnancy outcome [108].

Although its beneficial effect on pregnant sows has been confirmed by a large number of experiments, the supplementation of 0.8–1% arginine to the diet was reported to greatly increase the feed cost due to the high price of arginine, thereby restricting its wide use [109]. According to the summary of Table 1, low-dose arginine supplementation (about 0.1–0.4%) in sow diets during pregnancy can also promote pregnancy outcomes. Moreover, compared with the direct addition of arginine to the diet of sows, supplementation of NCG at the arginine level of 1/10 can have similar effects on their reproductive performance [106], thus reducing the high costs of direct supplementation of arginine to the diets of pregnant sows. In conclusion, the research and development of dietary supplementation strategies with low doses of arginine or NCG for pregnant sows will be a feasible way to improve the arginine metabolism, placental development, and fetal growth of sows.

## Conclusion

In the last decade, the selection for high prolificacy in modern sow herds has led to a marked increase in litter size. The consistent outcomes of this strategy are an increase of the embryonic losses during early gestation and the number of less vital and less mature low-birth-weight piglets at farrowing. The important contributors to this result seem to be aberrant placental development and functional impairment (deficits in vasculature) in early gestation and inadequate uterine capacity at all periods of gestation. Among nutrients, amino acids play the most important role in placental growth because they are absolutely required for synthesis of sufficient proteins to support the proliferation of trophectoderm and endothelial cells. Moreover, amino acids are not only the building blocks of proteins in cells, but also the precursors for synthesis of nitrogenous substances (e.g., NO, polyamines and glutathione) essential for placental growth and angiogenesis, conceptus development or scavenging free radicals to prevent placental oxidative damage. The requirements of amino acids in placentae differ markedly as gestation advances, but the amount of amino acids, especially glycine, proline, and arginine, is reduced due to the widespread practice of restricted feeding programs in sows during the entire gestation period to prevent excessive maternal body gains. Simply increasing the total crude protein content in the maternal diet is not an effective option because its oxidation generates high levels of ammonia and is extremely toxic to embryos and fetuses. Thus, adding specific amino acids that are deficient in the dietary supply is an attractive strategy to enhance placental growth and function, which in turn promotes fetal growth and development. As mentioned earlier, adding glycine, proline, arginine and their derivatives or endogenous synthesis promoters in the diets of pregnant sows will potentially promote the sow’s reproductive performance, fetal growth, and placental development. However, there is still a paucity of information on the effects of dietary functional amino acids on placental and conceptus development in swine except for arginine. This review has laid a theoretical basis for facilitating the translation of the basic research on glycine and proline biochemistry and physiology into feeding practice, and provided a reference for the nutritional strategy to improve the reproductive performance of sows.

## Data Availability

Not applicable.

## Abbreviations

BH_4_: Tetrahydrobiopterin
DFMO: Alpha-difluoromethylornithine
IUGR: Intrauterine growth retardation
mTOR: Mechanistic target of the rapamycin
NCG: N-carbamylglutamate
NO: Nitric oxide
NOSs: NO synthetases
Tie-2: Tunica interna endothelia cell kinase 2
VEGF: Vascular endothelial growth factor
